# The association between circulating lipoprotein subfractions and lipid content in coronary atheromatous plaques assessed by near-infrared spectroscopy

**DOI:** 10.1016/j.ijcha.2023.101215

**Published:** 2023-05-04

**Authors:** Julie Caroline Sæther, Elisabeth Kleivhaug Vesterbekkmo, Bruna Gigante, Guro Fanneløb Giskeødegård, Tone Frost Bathen, Turid Follestad, Rune Wiseth, Erik Madssen, Anja Bye

**Affiliations:** a Department of Circulation and Medical Imaging, Norwegian University of Science and Technology, Trondheim, Norway; b Clinic of Cardiology, St. Olavs Hospital, Trondheim, Norway; c National Advisory Unit on Exercise Training as Medicine for Cardiopulmonary Conditions, Trondheim, Norway; d Department of Medicine, Karolinska Institutet, Stockholm, Sweden; e Department of Public Health and Nursing, Norwegian University of Science and Technology, Trondheim, Norway; f Department of Clinical and Molecular Medicine, Norwegian University of Science and Technology, Trondheim, Norway; g Clinical Research Unit Central Norway, St. Olavs Hospital, Trondheim Norway

**Keywords:** NIRS, maxLCBI_4mm_, Coronary atherosclerosis, NMR spectroscopy, Lp(a), HDL subfractions, Lasso regression

## Abstract

**Background:**

Lipid content in coronary atheromatous plaques, measured by near-infrared spectroscopy (NIRS), can predict the risk of future coronary events. Biomarkers that reflect lipid content in coronary plaques may therefore improve coronary artery disease (CAD) risk assessment.

**Purpose:**

We aimed to investigate the association between circulating lipoprotein subfractions and lipid content in coronary atheromatous plaques in statin-treated patients with stable CAD undergoing percutaneous coronary intervention.

**Methods:**

56 patients with stable CAD underwent three-vessel imaging with NIRS when feasible. The coronary artery segment with the highest lipid content, defined as the maximum lipid core burden index within any 4 mm length across the entire lesion (maxLCBI_4mm_), was defined as target segment. Lipoprotein subfractions and Lipoprotein a (Lp(a)) were analyzed in fasting serum samples by nuclear magnetic resonance spectroscopy and by standard in-hospital procedures, respectively. Penalized linear regression analyses were used to identify the best predictors of maxLCBI_4mm_. The uncertainty of the lasso estimates was assessed as the percentage presence of a variable in resampled datasets by bootstrapping.

**Results:**

Only modest evidence was found for an association between lipoprotein subfractions and maxLCBI_4mm_. The lipoprotein subfractions with strongest potential as predictors according to the percentage presence in resampled datasets were Lp(a) (78.1 % presence) and free cholesterol in the smallest high-density lipoprotein (HDL) subfractions (74.3 % presence). When including established cardiovascular disease (CVD) risk factors in the regression model, none of the lipoprotein subfractions were considered potential predictors of maxLCBI_4mm_.

**Conclusion:**

In this study, serum levels of Lp(a) and free cholesterol in the smallest HDL subfractions showed the strongest potential as predictors for lipid content in coronary atheromatous plaques. Although the evidence is modest, our study suggests that measurement of lipoprotein subfractions may provide additional information with respect to coronary plaque composition compared to traditional lipid measurements, but not in addition to established risk factors. Further and larger studies are needed to assess the potential of circulating lipoprotein subfractions as meaningful biomarkers both for lipid content in coronary atheromatous plaques and as CVD risk markers.

## 1 Introduction

Lipid accumulation and inflammation in the coronary artery vessel wall are pivotal pathophysiological mechanisms in the development of coronary artery disease (CAD). It is demonstrated that the prognosis in CAD is strongly related to plaque geometry and composition [1]. Coronary plaques with a large lipid-rich core and an overlying thin fibrous cap are particularly vulnerable to rupture [2]. Intracoronary imaging studies using near-infrared spectroscopy (NIRS), a technique that enables identification of lipid content in coronary plaques [3], [4], have demonstrated that lipid content can predict the risk of future coronary events [5], [6], [7], [8]. NIRS is an invasive and resource-intensive procedure, and most often performed in conjunction with coronary angiography in symptomatic patients. Accordingly, it is of clinical interest to identify non-invasive surrogate biomarkers for lipid content in coronary plaques in order to improve risk stratification and optimal preventive treatment.

Lipoproteins, particularly low-density lipoprotein (LDL) cholesterol (LDL-C), play an essential role in atherosclerotic plaque initiation, progression, and composition [9], [10]. The risk for future cardiovascular events increases linearly with aggregated concentration of LDL-C, and the risk is also affected by the lifetime exposure of high concentrations [11]. Nevertheless, many patients with CAD have low cholesterol levels, and even with adequately lipid lowering treatment, the risk of future cardiovascular events remain significant [12], [13], [14].

Analysis of lipoprotein subfractions provides information of size, density, concentration, and compositions. It is suggested that small and dense LDL particles on cost of large and buoyant LDL particles result in a less favorable risk profile, and it is also questioned whether all high-density lipoprotein (HDL) subfractions hold atheroprotective properties [15], [16], [17]) . Furthermore, of increasing interest is Lipoprotein a (Lp(a)) which is an LDL-like particle with pro-atherosclerotic and pro-inflammatory properties. A causal continuous association between Lp(a) concentration and myocardial infarction has been demonstrated, even at low levels of LDL-cholesterol [18], [19], [20]. Measurements of lipoprotein subfractions may therefore improve risk stratification beyond traditional lipid measurements.

Studies assessing the association between circulating lipoproteins and lipid content in coronary atheromatous plaques, measured as the maximum lipid core burden index within any 4 mm length across the entire lesion (maxLCBI_4mm_), are sparse. To our knowledge, only traditional lipid measurements and Lp(a) have been investigated, with no or weak associations [21], [22], [23], [24]. Whether a more detailed lipoprotein subfraction analysis can identify new biomarkers with stronger associations to coronary plaque lipid content is unknown. In the present study, we aimed to investigate the association between circulating lipoprotein subfractions and lipid content in coronary atheromatous plaques, measured as maxLCBI_4mm_ by NIRS, in patients with stable CAD undergoing percutaneous coronary intervention (PCI).

## 2 Methods

### 2.1 Study design and ethics

This cross-sectional study was based on baseline data from the *Impact of Cardiac Exercise Training on Lipid Content in Coronary Atheromatous Plaques Evaluated by Near-Infrared Spectroscopy* (CENIT) [25]. The study was approved by the regional ethics committee of central Norway (2015:210), registered at clinicaltrials.gov (NCT02494947), and conducted in accordance with the Declaration of Helsinki. Written and informed consent was obtained from all participants, and their personal information was handled and stored with high security in accordance with laws and regulations.

### 2.2 Study participants

Patients diagnosed with a hemodynamic significant coronary artery stenosis in at least one epicardial vessel that required PCI were screened for inclusion between February 2016 and April 2019 at St. Olavs Hospital in Trondheim, Norway. Inclusion criteria was stable statin therapy for at least six weeks prior to the angiographic examination to avoid anti-atherosclerotic effects following initiation of high-dose statin therapy and for stabilization of the circulating lipid profile [26], [27]. Exclusion criteria were prior coronary artery bypass graft surgery and known inflammatory disease (other than atherosclerosis). A total of 60 eligible patients gave written informed consent to participate in the study.

### 2.3 Intracoronary imaging

Following stent implantation and intracoronary administration of 200 µg nitroglycerine, three-vessel intracoronary imaging was performed when feasible to quantify lipid content in non-culprit coronary plaques. The near-infrared spectroscopy intravascular ultrasound (NIRS-IVUS) catheter (TVC-MC8 model system with a 3.2Fr 40 MHz catheter, Infraredx, Burlington, MA) was positioned as distal as possible in the coronary artery and pulled back to the ostium or the guiding catheter at a speed of 0.5 mm/s. Intracoronary imaging data and angiograms were analyzed with a commercial software (Pie Medical Imaging Software, CAAS Intravascular) at an independent core facility (KCRO, Krakow, Poland) blinded for patient characteristics. The stented segment with its corresponding 5 mm edge segments in both directions was excluded from the analysis.

**Fig. 1:**
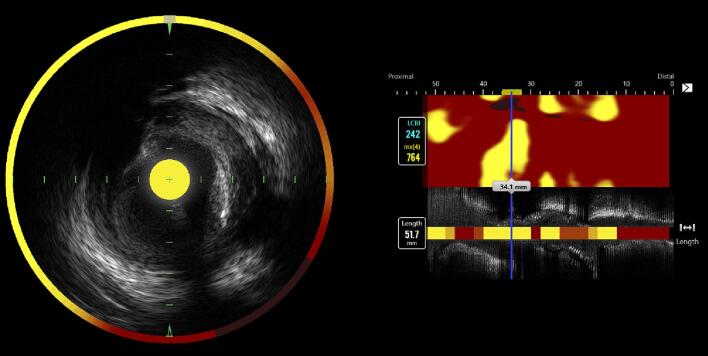
Left anterior descending artery imaged with combined near‐infrared spectroscopy and intravascular ultrasound (NIRS‐IVUS) catheter. To the left, a cross-sectional image with a color-coded circumflex that illustrates lipid accumulation within the plaque. Yellow represents high probability of lipids and red represents no lipid. The NIRS-derived chemogram to the right illustrates the maximum lipid core burden index within any 4 mm segment across the entire lesion of 764 (76.4 %).

The lipid core burden index ranges from 0 to 1000 and was calculated from a NIRS derived chemogram with color coded pixels (Fig. 1). The colors span from red to yellow with increased probability of lipid-rich plaques [3]. The coronary artery segment with the highest measured lipid content, defined as the maximum lipid core burden index (range between 0 and 1000) within any 4 mm segment length across the entire lesion (maxLCBI_4mm_), was considered the most diseased segment and thus defined as target segment.

### 2.4 Data collection

Following standard in-hospital procedures, fasting venous blood samples were collected early in the morning the day after PCI. Within 1 hour, a 5 mL serum tube with clot activator was centrifuged (Rotina 420R, Hettich zentrifugen) at 3000 × *g* for 10 min at room temperature (20 °C). The sample was further aliquoted into microfuge tubes, marked, and stored in a biobank at −80 °C until nuclear magnetic resonance (NMR) spectroscopy analysis. In addition, total cholesterol, HDL cholesterol (HDL-C), LDL-C, triglycerides, Apolipoprotein-A1 (Apo-A1), Apolipoprotein-B (Apo-B), Lp(a), creatinine, hemoglobin, and glycated hemoglobin A1c were analyzed in blood samples using standard in-hospital procedures at the Department of Medical Biochemistry, St. Olavs Hospital. In our study, Lp(a) were categorized into elevated Lp(a), defined as Lp(a) > 30 mg/dL, and normal Lp(a), defined as Lp(a) < 30 mg/dL. Information about age (years), body mass index (calculated as kg·m^−2^), blood pressure, smoking status, diabetes mellitus, medication use, comorbidities, medically treated hypertension, hyperlipidemia, previous cardiovascular disease (CVD), and heredity for CVD were collected from the hospital medical records at time of inclusion. Medically treated hypertension and hyperlipidemia were defined as patients previously diagnosed with these conditions by a general practitioner or in an outpatient clinic. Previous CVD was defined as patients with previous CAD, stroke, peripheral arterial disease, and/or aortic disease, and heredity of CVD was defined as father or mother with CVD before the age of 55 years and 65 years, respectively.

### 2.5 Lipoprotein subfraction analysis by nuclear magnetic resonance spectroscopy

NMR spectroscopy was performed using a Bruker Avance III Ultrashield Plus 600 MHz spectrometer (Bruker BioSpin, GmBH, Rheinstetten, Germany) equipped with a 5 mm QCI Cryoprobe at the MR Core Facility, NTNU. Buffer (150 μl, 20 % D_2_O with 0.075 M Na_2_HPO_4_, 6 mM NaN_3_, 4.6 mM trimethylsilylpropanoic acid (TSP), pH 7.4) was mixed with 150 μl thawed serum and transferred to 3 mm NMR tubes. Further procedures were fully automated using a SampleJet with Icon-NMR on Topspin 3.1 (Bruker BioSpin). 1D 1H Nuclear Overhauser effect spectroscopy (NOESY) and Carr-Purcell-Meiboom-Gill (CPMG) spectra with water presaturation was obtained at 310 K. The spectra were Fourier transformed to 128 K after 0.3 Hz exponential line broadening.

An automated Bruker IVDr Lipoprotein Subclass Analysis (B.I.LISA^TM^) was used to quantify 114 lipid variables, where 106 of these variables were considered as lipoprotein subfractions [28] (Appendix 1). In total serum, the concentration of cholesterol, triglycerides, Apo-A1, Apolipoprotein-A2 (Apo-A2), and Apo-B/particle number were measured. The ratios LDL-C/HDL-C and Apo-B/Apo-A1 were further calculated. Also, concentrations of cholesterol, free cholesterol, phospholipids, and triglycerides were measured in LDL, HDL, intermediate-density lipoprotein (IDL) and very-low-density lipoprotein (VLDL)), and in their 15 size-based subfractions (LDL 1–6, VLDL 1–5 and HDL 1–4). In addition, Apo-B/particle number was measured in LDL, IDL, VLDL, and LDL 1–6. Apo-A1 and Apo-A2 were measured in HDL and HDL 1–4. With increasing number from 1 to 6 in LDL, 1 to 5 in VLDL, and 1 to 4 in HDL, the particle size decreases. The density ranges of lipoproteins and lipoprotein subfractions are included in Appendix 2**,** and the median with 25- and 75 percentiles for each NMR-derived lipid variable in the study population are included in Appendix 1.

### 2.6 Statistical analyses

The data was analyzed by IBM SPSS Statistics (version 27.0, Armonk, NY: IBM Corp) and R (version 4.0.2). All continuous data are presented as median and inter quartile range and categorical data as frequencies with percentages unless stated otherwise. Lipid variables were assessed for normality by the Shapiro-Wilk test and visual inspection of normal QQ plots. As appropriate, Pearson or Spearman correlations were used to evaluate the correlation between each lipid variable and maxLCBI_4mm_. To estimate the effective number of independent tests for multiple testing correction, principal component analysis was used. Nine principal components explained > 95 % of the variance, and the corrected threshold for assessing statistical significance was therefore 0.05/9 = 0.005 (p < .005). The rationale for this method has been described previously and applied in several metabolic profile studies [29], [30].

To study the joint association between lipid variables and maxLCBI_4mm_, the least absolute shrinkage and selection operator (lasso) method for penalized linear regression, as implemented in the *glmnet* package [31] in R, was used. In a model with many predictors, penalized regression aims to reduce the complexity of the model by imposing a penalty, so that the regression coefficients for variables with low predictive value are shrunken towards zero. The method can simultaneously perform parameter estimation and variable selection, as some variables are shrunken to exactly zero. The degree of shrinkage was determined by ten times 10-fold cross-validation, minimizing the mean square error. The uncertainty of the estimated coefficients from the lasso was assessed by bootstrapping. The fitting procedure was repeated for 1000 bootstrap samples, and the uncertainty for each variable was represented by the proportion of the bootstrap samples for which its coefficient was not set to zero in the estimated model. The model was fitted to two sets of predictors: one model with lipoprotein subfractions, including Lp(a) (N = 107), and one model including both lipoprotein subfractions and 14 established risk factors for CVD (N = 121). The CVD risk factors includes total cholesterol, total triglycerides, LDL-C, HDL-C, LDL-C/HDL-C, Apo-B/Apo-A1, age, body mass index, smoking, diabetes mellitus, medically treated hypertension, hyperlipidemia, previous CVD, and heredity of CVD. All variables were continuous, except from Lp(a) (above/beneath 30 mg/dL), smoking (yes/no), diabetes (yes/no), medically treated hypertension (yes/no), hyperlipidemia (yes/no), previous CVD (yes/no), and heredity of CVD (yes/no).

In the main results, the lipoprotein subfractions and risk factors for CVD that were included in > 50 % of the bootstrap samples and had a non-zero regression coefficient were included in figures and tables. Additional results are presented in appendixes.

## 3 Results

Out of the 60 patients enrolled in the CENIT-study, this post-hoc analysis included 56 eligible patients with both evaluable NMR spectroscopy data and NIRS-derived maxLCBI_4mm_ data (Appendix 3). Patient characteristics and NIRS-IVUS derived plaque characteristics for the targeted segments are presented in Table 1**.** NMR measurements of Apo-1, Apo-B, triglycerides, HDL-C, LDL-C, and total cholesterol were compared with gold-standard laboratory measurements to assess the internal validity of NMR spectroscopy. The internal validity was found to be high (Appendix 4).

**Table 1:** Patient characteristics and NIRS-IVUS derived plaque characteristics for the study population (N = 56).

**Variables**	**Total, N = 56**
**General**	
Age, years	57.5 (52.0–65.0)
Males, (n, %)	53 (94.6 %)
Body mass index, kg·m^−2^	28.0 (26.1–31.0)
Smoking, current and ex-smoker (n, %)	34 (60.7 %)
Systolic blood pressure, mmHg	143.0 (133.2–151.0)
Diastolic blood pressure, mmHg	84.5 (80.0–89.0)
**Medical history**	
Diabetes mellitus (n, %)	7 (12 %)
Hypertension (n, %)	30 (54 %)
Hyperlipidemia (n, %)	19 (34 %)
Heredity of premature cardiovascular disease (n, %)	47 (84 %)
Prior history of cardiovascular disease (n, %)	27 (48 %)
**Medication**	
Statins (n, %)	56 (100 %)
Dual antiplatelet therapy (n, %)	56 (100 %)
Combined therapy with Ezetimibe (n, %)	2 (4 %)
Diuretics (n, %)	6 (10.7 %)
Calcium blockers (n, %)	11 (19.6 %)
Betablockers (n, %)	20 (36 %)
ACE inhibitors/angiotensin II receptor antagonists (n, %)	29 (52 %)
**Clinical measurements**	
Total cholesterol, mmol/L	3.5 (3.2–4.1)
Low-density lipoprotein cholesterol, mmol/L	2.0 (1.7–2.5)
High-density lipoprotein, mmol/L	1.0 (0.9–1.1)
Triglycerides, mmol/L	1.3 (1.0–1.7)
Apolipoprotein A1, g/L	1.3 (1.2–1.4)
Apolipoprotein B, g/L	0.7 (0.6–0.8)
Lipoprotein a, mg/L	147.0 (100–811)
Creatinine, µmol/L	79.0 (72.0–88.5)
Hemoglobin, g/dL	14.8 (14.0–15.4)
Glycosylated hemoglobin, %	5.5 (5.2–5.9)
**Target segments**	
Left anterior descending artery (n, %)	24 (43 %)
Circumflex artery (n, %)	13 (23 %)
Right coronary artery (n, %)	19 (34 %)
**NIRS-IVUS derived plaque characteristics for the targeted segments**	
maxLCBI_4mm_	326.0 (172.5–402.5)
Total lipid core burden index at region of interest	111.5 (39.0–182.5)
Plaque burden, %	49.2 (42.6–54.8)
Minimal lumen area, mm^2^	4.8 (3.9–6.6)
Stenosis at minimal lumen area, %	64.2 (55.1–73.6)
Plaque volume, mm^3^	155.5 (93.5–261.7)
Vessel volume, mm^3^	292.3 (191.0–469.6)
Lumen volume, mm^3^	154.2 (102.1–231.4)
Segment length, mm	20.5 (14.3–29.2)

Data are presented as median with 25 and 75 percentiles or numbers with percentages. NIRS-IVUS, near-infrared spectroscopy intravascular ultrasound; ACE inhibitors, Angiotensin-converting-enzyme inhibitors; maxLBI_4mm_, the maximum lipid core burden index within any 4 mm segment across the entire lesion; NIRS-IVUS, near-infrared spectroscopy intravascular ultrasound.

### 3.1 MaxLCBI_4mm_ and lipoprotein subfractions

The Spearman correlations between maxLCBI_4mm_ and each of the lipid variables were in the range from −0.293 to 0.196, and none were statistically significant after adjustment for multiple testing, with p-values from 0.028 to 0.991 (Appendix 5). Cholesterol in the smallest VLDL subfractions, VLDL-5, was the lipoprotein subfraction most strongly correlated with maxLCBI_4mm_ (corr. coeff = −0.293, p = .028).

**Fig. 2:**
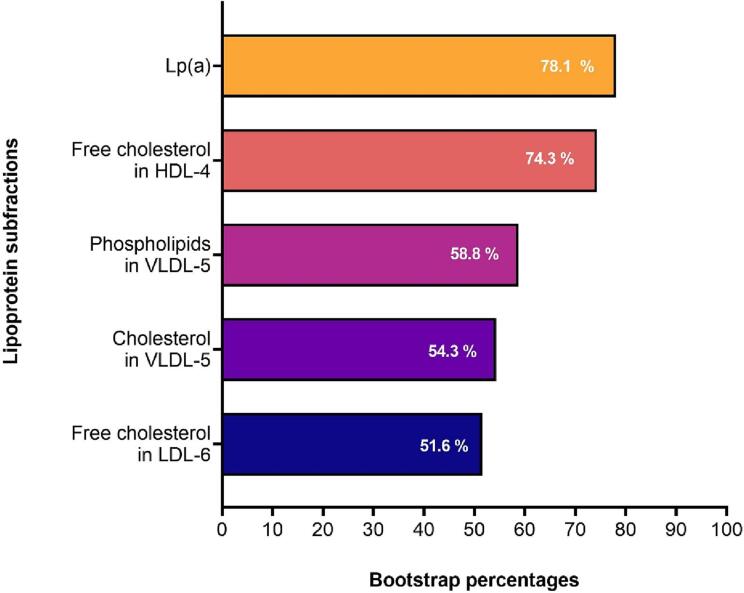
Lipoprotein subfractions that had the strongest potential as predictors for maxLCBI_4mm_ according to the percentage presence in the resampled datasets by bootstrapping. The presented lipoprotein subfractions were present in >50 % of the resampled dataset and had a non-zero regression coefficient from lasso. Lp(a), lipoprotein a; HDL-4, high-density lipoprotein 4; VLDL-5; very-low-density lipoprotein 5; LDL-6, low-density lipoprotein 6; Lasso, least absolute shrinkage, and selection operator.

In the multivariable analysis, Lp(a) and free cholesterol in the smallest HDL subfractions, HDL-4, were the lipoproteins most strongly associated with maxLCBI_4mm_ according to the percentage presence in the resampled datasets that included each lipoprotein subfraction (Fig. 2). Lp(a) was included in 78.1 % of the resampled dataset, and patients with elevated Lp(a) (n = 23) had an estimated average of 57.0 unit higher maxLCBI_4mm_ values than patients with normal Lp(a) levels (n = 33, Table 2). Free cholesterol in HDL-4 was included in 74.3 % of the resampled dataset, and the maxLCBI_4mm_ increased with an estimated average of 36.5 units for every unit increase (mg/dL) of free cholesterol in HDL-4 (Table 2). Extended results, including all analyzed lipoproteins are listed in Appendix 6.

**Table 2:** The estimated lasso regression coefficients of the lipoprotein subfractions that were present in > 50 % of the resampled datasets by bootstrapping.

**Lipoprotein subfractions**	**Regression coefficient**
Lp(a)	57.0
Free cholesterol in HDL-4	36.5
Phospholipids in VLDL-5	−78.5
Cholesterol in VLDL-5	–32.5
Free cholesterol in LDL-6	21.9

Lasso, least absolute shrinkage, and selection operator; Lp(a), lipoprotein a; HDL-4, high-density lipoprotein 4; VLDL-5, very-low-density lipoprotein 5; LDL-6, low-density lipoprotein 6.

### 3.2 MaxLCBI_4mm_, lipoprotein subfractions, and CVD risk factors

When including established CVD risk factors in the regression model, the association between maxLCBI_4mm_ and both Lp(a) and free cholesterol in HDL-4 was weakened. Lp(a) and free cholesterol in HDL-4 were included in 67.1 % and 44.6 % of the resampled datasets by bootstrapping, respectively, and had an estimated regression coefficient of zero (Appendix 7).

Among the CVD risk factors, medically treated hypertension was included in 91.7 % of the resampled datasets and had a regression coefficient of −49.5 unit, meaning that patients that were medically treated for hypertension had on average 49.5 units lower maxLCBI_4mm_ compared to patients not medically treated for hypertension (Appendix 7). None of the traditional lipid measurements were found to be potential predictors of maxLCBI_4mm_ as they had a regression coefficient of zero and were included in less than 11 % of the resampled datasets (Table 3).

**Table 3:** The percentage presence of the traditional lipid measurements in the resampled datasets by bootstrapping.

**Clinical biomarkers**	**Percentage included**
LDL-C/HDL-C	10.2
HDL-C (mg/dL)	3.7
Total cholesterol (mg/dL)	2.5
Apo-B/Apo-A1	2.1
LDL-C (mg/dL)	1.3
Total triglycerides (mg/dL)	0

Percent included, how frequently the traditional lipid measurements were included in the model across the 1000 bootstrap samples; LDL-C, low-density lipoprotein cholesterol; HDL-C, high-density lipoprotein cholesterol; Apo-B/Apo-A1, apolipoprotein B/apolipoprotein A1.

## 4 Discussion

In this study, we investigated the association between circulating lipoprotein subfractions and lipid content in coronary atheromatous plaques in statin-treated patients with stable CAD undergoing PCI. The main findings were: 1) Lp(a) and free cholesterol in the smallest HDL subfractions, HDL-4, were the lipoprotein subfractions with the strongest potential as predictors of coronary lipid content measured as maxLCBI_4mm_, 2) after including established CVD risk factors in the regression model, the association between coronary lipid content and both Lp(a) and free cholesterol in HDL-4 was weakened, and 3) we did not detect any associations between traditional lipid measurements and coronary lipid content.

To the best of our knowledge, this is the first study to investigate the association between a large number of lipoprotein subfractions and lipid content in coronary atheromatous plaques measured as maxLCBI_4mm_ by NIRS. Although hyperlipidemia, and particularly high levels of LDL-C, is considered a major risk factor for CVD, only a few studies have assessed the correlation or association between coronary lipid content measured as maxLCBI_4mm_ and traditional lipid measurements in patients with CVD [21], [22], [23], [24]. One study demonstrated that the percent change in HDL-C was negatively associated with the percent change in maxLCBI_4mm_ after 13 months follow-up in patients with acute coronary syndrome or stable CAD [22]. Another study found a negative correlation between HDL-C and maxLCBI_4mm_ in patients with acute coronary syndrome or stable CAD with a maxLCBI_4mm_ ≥ 323, but not in patients with a maxLCBI_4mm_ < 323 [21]. In these two studies, 78 % and 85.7 % of the patients were on statin-therapy, respectively. In statin-treated patients with CAD, an association between LDL-C and maxLCBI_4mm_ was recently demonstrated, while other traditional lipid measurements were not included in this analysis [23]. Furthermore, a post-hoc analysis of the Lipid Rich Plaque Study that included 984 patients, investigated the potential correlation between maxLCBI_4mm_ and both LDL- and HDL-C, and did not detect any significant correlations among statin-naïve patients, statin-treated patients, or in the total population [24]. This is in line with the present study, as none of the traditional lipid measurements were associated with lipid content in coronary atheromatous plaques. This may be due to the effect of lipid-lowering therapy as all patients were on stable statin-treatment for at least 6 weeks prior to inclusion. Statins are known to reduce LDL, VLDL and IDL cholesterol, and slightly increase HDL-C and Lp(a), and to provide positive effects on coronary lipid content and plaque stabilization [26]. Lp(a) and free cholesterol in HDL-4 were the lipoprotein subfractions most strongly associated with coronary lipid content in our study, suggesting that these lipoprotein subfractions may not be substantially affected by statins and may provide additional information with respect to coronary plaque composition compared to traditional lipid measurements.

Genetic and observational evidence has convincingly demonstrated a causal and linear relationship between high concentrations of Lp(a) and atherosclerotic CVD and cardiovascular- and all-cause mortality [20], [32], [33], [34]) . Lp(a) levels are slightly increased by statins, but several *meta*-analyses support that the statin-induced changes in Lp(a) levels are not clinically significant [35], [36], [37]. Thus, statins are not considered to change the Lp(a)-associated risk of CVD. In our study, Lp(a) were categorized into elevated Lp(a), defined as Lp(a) > 30 mg/dL, and normal Lp(a), defined as Lp(a) < 30 mg/dL, which was the most used approach at the time of inclusion [34]. We found that Lp(a) was the lipoprotein most strongly associated with maxLCBI_4mm_, suggesting that high levels of Lp(a) may predict coronary lipid content in patients with stable CAD. A study by Nakamura et al. [23] recently demonstrated that Lp(a) was associated with maxLCBI_4mm_ in patients with both CAD and diabetes, while not in patients with CAD and no diabetes. Lp(a) is considered an important promoter of plaque vulnerability, partly by binding to oxidative phospholipids with pro-inflammatory properties and housing the glycation of Apo-B [38], mechanisms which are known to be increased in diabetic patients. Another study using optical coherence tomography found that patients with Lp(a) > 30 mg/dL had more high-risk plaques, which included more lipid-rich plaques and thinner cap fibroatheromas, and wider lipid arcs, compared to patients with Lp(a) < 30 mg/dL [39].

HDL is generally known to be negatively associated with coronary atherosclerosis, but a causal association between HDL-C and CVD has been challenged by large Mendelian randomization studies and HDL-C raising drug trials [40], [41]. Since HDL is highly heterogeneous, it is suggested that not all HDL subfractions holds atheroprotective properties and that total HDL-C is not a sufficient measure of its protective properties. To the best of our knowledge, no previous study has investigated the association between lipid content in coronary artery plaque, measured as maxLCBI_4mm_ by NIRS, and HDL subfractions in patients with CAD, nor the association between HDL subfractions and cardiovascular outcomes. Even though we did not detect an association between lipid content in coronary plaque and serum HDL-C, we found free cholesterol in the smallest HDL subfractions, HDL-4, to be one of the lipoprotein subfractions most strongly associated with maxLCBI_4mm_. The evidence of free cholesterol in HDL-4 as a potential predictor of lipid content was however substantially reduced after including established risk factors in the regression model. Although the role of particle size remains controversial, our study supports the assumptions that not all HDL subfractions necessarily have atheroprotective properties, that the smallest subfractions may increase the CVD risk, and that HDL subfractions may add information beyond total HDL-C. Since statins only induce a small increase in HDL-C, HDL subfractions may not be substantially affected, and could therefore represent valuable markers of coronary lipid content in statin-treated patients with stable CAD.

In the regression analysis that included CVD risk factors and lipoprotein subfractions, patients with medically treated hypertension had on average 49.5 units lower maxLCBI_4mm_ compared to patients without known hypertension. In our study, hypertension that required medical treatment was diagnosed prior to inclusion. Accordingly, these patients may have been offered more intensive preventive therapy with influence on plaque lipid content.

There are some limitations to address. First, our sample size was limited. Our study included 53 males and 3 females, and since the lipoprotein subfraction profile may be sex-specific [42], [43], our results may only be applicable for males. Secondly, PCI was a requirement for inclusion, and for ethical reasons, blood samples were taken after PCI when patients were found eligible. The vessel trauma induced by PCI may have affected the lipoprotein subfraction profile. In addition, all patients had to be on stable statin treatment for at least 6 weeks prior to inclusion, which influence the lipoprotein subfraction profile. However, for lipoprotein subfractions to be clinically useful as a biomarker for CVD risk, it should also be applicable to patients on statins. A strength in our study is that multivessel imaging was conducted to ensure detection of the most diseased vessel. MaxLCBI_4mm_ was used to target the most diseased coronary segment, but this is not necessarily representative for the total atherosclerotic burden. Another strength is that all NIRS-IVUS data were analyzed at an independent core facility.

## 5 Conclusion

In this study of lipoprotein subfractions and coronary lipid content, Lp(a) and free cholesterol in the smallest HDL subfractions, HDL-4, had the highest potential as predictors of coronary lipid content in statin-treated patients with stable CAD. However, only moderate evidence was demonstrated, and adjusting for established risk factors for CVD weakened the associations. Further and larger studies are needed to assess the potential of circulating lipoprotein subfractions as meaningful markers both for lipid content in coronary atheromatous plaques and as CVD risk markers.

## 6 Sources of funding

The study was supported by grants from the Norwegian Health Association, The Liaison Committee for education, research and innovation in Central Norway, and the Joint Research Committee between St. Olavs hospital and the Faculty of Medicine and Health Sciences, the Norwegian University of Science and Technology (FFU).

## Declaration of Competing Interest

The authors declare that they have no known competing financial interests or personal relationships that could have appeared to influence the work reported in this paper.

## Figures and Tables

**Table:** Lipid variables (N = 114) with its matrix, analyte, and unit analyzed by nuclear magnetic resonance spectroscopy, and the calculated median and percentiles for each lipid variable in our study population (N = 56).

**Lipid variables**	**Unit**	**Median (25–75 percentiles)**
Total Plasma Triglycerides*	mg/dL	118.6 (88.5–162.4)
Total Plasma Cholesterol*	mg/dL	137.6 (122.7–165.5)
LDL Cholesterol*	mg/dL	68.4 (56.9–84.6)
HDL Cholesterol*	mg/dL	40.7 (36.4–45.0)
Total Plasma Apo-A1	mg/dL	111.9 (105.3–119.4)
Total Plasma Apo-A2	mg/dL	22.5 (20.6–24.8)
Total Plasma Particle Number	nmol/L	1140.6 (1013.9–1414.7)
Total Plasma Apo-B	mg/dL	62.7 (55.8–77.8)
LDL cholesterol/HDL cholesterol*	–	1.6 (1.4–2.1)
Apo-B/Apo-A1*	–	0.6 (0.5–0.72)
VLDL Particle Number	nmol/L	153.8 (124.7–210.4)
IDL Particle Number	nmol/L	46.3 (25.6–75.4)
LDL Particle Number	nmol/L	913.9 (745.5–1117.4)
LDL-1 Particle Number	nmol/L	144.3 (128.1–179.7)
LDL-2 Particle Number	nmol/L	102.4 (77.7–128.2)
LDL-3 Particle Number	nmol/L	111.1 (74.5–147.5)
LDL-4 Particle Number	nmol/L	107.4 (58.1–158.2)
LDL-5 Particle Number	nmol/L	131.1 (93.1–195.6)
LDL-6 Particle Number	nmol/L	278.4 (236.1–367.2)
VLDL Triglycerides	mg/dL	86.0 (60.4–117.3)
IDL Triglycerides	mg/dL	10.1 (4.8–17.8)
LDL Triglycerides	mg/dL	13.2 (10.3–16.3)
HDL Triglycerides	mg/dL	8.5 (7.4–9.9)
VLDL Cholesterol	mg/dL	15.4 (11.2–23.7)
IDL Cholesterol	mg/dL	4.5 (1.2–10.3)
LDL Cholesterol†	mg/dL	68.4 (56.9–84.6)
HDL Cholesterol†	mg/dL	40.7 (36.4–45.0)
VLDL Free Cholesterol	mg/dL	9.2 (7.3–12.8)
IDL Free Cholesterol	mg/dL	1.0 (0.2–2.8)
LDL Free Cholesterol	mg/dL	23.9 (20.7–28.9)
HDL Free Cholesterol	mg/dL	11.9 (10.4–13.7)
VLDL Phospholipids	mg/dL	21.5 (16.4–29.5)
IDL Phospholipids	mg/dL	2.9 (1.4–6.3)
LDL Phospholipids	mg/dL	41.8 (35.1–50.3)
HDL Phospholipids	mg/dL	53.8 (49.4–60.3)
HDL Apo-A1	mg/dL	109.9 (103.2–118.4)
HDL Apo-A2	mg/dL	23.4 (21.4–25.9)
VLDL Apo-B	mg/dL	8.5 (6.9–11.6)
IDL Apo-B	mg/dL	2.5 (1.4–4.1)
LDL Apo-B	mg/dL	50.3 (41.0–61.4)
VLDL-1 Triglycerides	mg/dL	47.2 (33.2–66.9)
VLDL-2 Triglycerides	mg/dL	14.6 (10.3–20.2)
VLDL-3 Triglycerides	mg/dL	11.0 (8.1–16.2)
VLDL-4 Triglycerides	mg/dL	8.3 (7.3–11.3)
VLDL-5 Triglycerides	mg/dL	3.6 (3.3–4.1)
VLDL-1 Cholesterol	mg/dL	5.7 (3.8–9.1)
VLDL-2 Cholesterol	mg/dL	1.9 (1.1–3.1)
VLDL-3 Cholesterol	mg/dL	1.9 (1.0–3.6)
VLDL-4 Cholesterol	mg/dL	3.2 (2.3–5.3)
VLDL-5 Cholesterol	mg/dL	1.6 (1.4–1.9)
VLDL-1 Free Cholesterol	mg/dL	2.2 (1.2–3.7)
VLDL-2 Free Cholesterol	mg/dL	1.0 (0.6–1.5)
VLDL-3 Free Cholesterol	mg/dL	1.1 (0.7–2.0)
VLDL-4 Free Cholesterol	mg/dL	1.2 (0.8–2.2)
VLDL-5 Free Cholesterol	mg/dL	0.7 (0.5–0.9)
VLDL-1 Phospholipids	mg/dL	6.9 (4.1–10.0)
VLDL-2 Phospholipids	mg/dL	3.2 (2.0–4.4)
VLDL-3 Phospholipids	mg/dL	3.1 (2.1–4.9)
VLDL-4 Phospholipids	mg/dL	3.6 (3.0–5.2)
VLDL-5 Phospholipids	mg/dL	2.3 (2.0–2.5)
LDL-1 Triglycerides	mg/dL	4.5 (3.7–5.7)
LDL-2 Triglycerides	mg/dL	1.3 (0.9–1.7)
LDL-3 Triglycerides	mg/dL	2.1 (1.8–2.4)
LDL-4 Triglycerides	mg/dL	1.2 (0.8–1.8)
LDL-5 Triglycerides	mg/dL	1.4 (0.9–1.9)
LDL-6 Triglycerides	mg/dL	4.4 (3.7–5.8)
LDL-1 Cholesterol	mg/dL	12.5 (10.1–16.1)
LDL-2 Cholesterol	mg/dL	8.4 (5.9–11.3)
LDL-3 Cholesterol	mg/dL	9.0 (5.5–13.1)
LDL-4 Cholesterol	mg/dL	9.5 (5.7–13.5)
LDL-5 Cholesterol	mg/dL	10.1 (7.4–14.7)
LDL-6 Cholesterol	mg/dL	19.3 (16.1–25.4)
LDL-1 Free Cholesterol	mg/dL	3.8 (3.1–4.7)
LDL-2 Free Cholesterol	mg/dL	2.9 (2.1–3.8)
LDL-3 Free Cholesterol	mg/dL	3.5 (2.5–4.5)
LDL-4 Free Cholesterol	mg/dL	3.3 (2.6–4.4)
LDL-5 Free Cholesterol	mg/dL	3.2 (2.6–4.1)
LDL-6 Free Cholesterol	mg/dL	4.9 (4.2–6.1)
LDL-1 Phospholipids	mg/dL	7.9 (6.9–9.8)
LDL-2 Phospholipids	mg/dL	5.3 (3.9–6.8)
LDL-3 Phospholipids	mg/dL	5.6 (3.6–7.6)
LDL-4 Phospholipids	mg/dL	5.8 (3.9–7.9)
LDL-5 Phospholipids	mg/dL	5.8 (4.5–8.0)
LDL-6 Phospholipids	mg/dL	10.5 (8.8–13.7)
LDL-1 Apo-B	mg/dL	7.9 (7.0–9.9)
LDL-2 Apo-B	mg/dL	5.6 (4.3–7.1)
LDL-3 Apo-B	mg/dL	6.1 (4.1–8.1)
LDL-4 Apo-B	mg/dL	5.9 (3.2–8.7)
LDL-5 Apo-B	mg/dL	7.2 (5.1–10.8)
LDL-6 Apo-B	mg/dL	15.3 (13.0–20.2)
HDL-1 Triglycerides	mg/dL	2.0 (1.6–2.7)
HDL-2 Triglycerides	mg/dL	1.2 (1.0–1.5)
HDL-3 Triglycerides	mg/dL	1.6 (1.4–1.9)
HDL-4 Triglycerides	mg/dL	3.4 (2.9–4.0)
HDL-1 Cholesterol	mg/dL	9.9 (7.8–13.0)
HDL-2 Cholesterol	mg/dL	4.6 (3.3–5.4)
HDL-3 Cholesterol	mg/dL	6.8 (6.0–7.5)
HDL-4 Cholesterol	mg/dL	19.2 (17.3–20.7)
HDL-1 Free Cholesterol	mg/dL	2.7 (1.9–3.2)
HDL-2 Free Cholesterol	mg/dL	1.1 (0.8–1.5)
HDL-3 Free Cholesterol	mg/dL	1.8 (1.4–2.1)
HDL-4 Free Cholesterol	mg/dL	4.0 (3.5–4.5)
HDL-1 Phospholipids	mg/dL	10.4 (8.2–13.6)
HDL-2 Phospholipids	mg/dL	7.5 (5.4–8.6)
HDL-3 Phospholipids	mg/dL	11.1 (9.5–12.4)
HDL-4 Phospholipids	mg/dL	25.4 (23.4–27.8)
HDL-1 Apo-A1	mg/dL	8.5 (6.2–13.5)
HDL-2 Apo-A1	mg/dL	11.6 (10.3–13.4)
HDL-3 Apo-A1	mg/dL	19.9 (17.8–22.0)
HDL-4 Apo-A1	mg/dL	70.7 (66.5–77.3)
HDL-1 Apo-A2	mg/dL	0.5 (0.1–1.0)
HDL-2 Apo-A2	mg/dL	1.3 (0.8–1.8)
HDL-3 Apo-A2	mg/dL	3.8 (3.3–4.2)
HDL-4 Apo-A2	mg/dL	17.4 (15.8–19.3)

**Table:** Density ranges of lipoproteins and lipoprotein subfractions.

	**Ranges, kg/L**
Main lipoprotein fractions	
LDL	1.019–1.063
VLDL*	0.950–1.006
IDL	1.006–1.019
HDL	1.063–1.210
Low-density lipoprotein subfractions	
LDL-1	1.019–1.031
LDL-2	1.031–1.034
LDL-3	1.034–1.037
LDL-4	1.037–1.040
LDL-5	1.040–1.044
LDL-6	1.044–1.063
High-density lipoprotein subfractions	
HDL-1	1.063–1.100
HDL-2	1.100–1.112
HDL-3	1.112–1.125
HDL-4	1.125–1.210

**Table:** The Spearman correlation between lipid variables from nuclear magnetic resonance (NMR) spectroscopy and lipid measurements from gold-standard laboratory measurements, indicating high internal validity.

**Gold-standard laboratory measurements**	**Unit**	**NMR spectroscopy measurements**	**Unit**	**Spearman correlation** **coefficient**	**p-value**	**Number**
Apolipoprotein A1	g/L	Apolipoprotein A1	mg/dL	0.851	<0.001	56
Apolipoprotein B	g/L	Apolipoprotein B	mg/dL	0.921	<0.001	56
Total triglycerides	mmol/L	Total triglycerides	mg/dL	0.944	<0.001	56
HDL-C	mmol/L	HDL-C	mg/dL	0.874	<0.001	56
LDL-C	mmol/L	LDL-C	mg/dL	0.838	<0.001	56
Total cholesterol	mmol/L	Total cholesterol	mg/dL	0.937	<0.001	56

**Table:** Spearman correlations between maxLCBI_4mm_ and lipid variables (N = 112).

**Lipoprotein variables**	**Spearman correlation coefficient**	**p-value**	**Number**
Total Plasma Triglycerides	−0.107	0.431	56
Total Plasma Cholesterol	−0.030	0.825	56
LDL Cholesterol	−0.052	0.702	56
HDL Cholesterol	0.099	0.466	56
Total Plasma Apo-A1	0.069	0.615	56
Total Plasma Apo-A2	0.162	0.234	56
Total Plasma Apo-B	−0.058	0.671	56
LDL cholesterol/HDL cholesterol	−0.071	0.605	56
Apo-B/Apo-A1	−0.062	0.652	56
Total Plasma Particle Number	−0.058	0.671	56
VLDL Particle Number	−0.114	0.404	56
IDL Particle Number	−0.060	0.661	56
LDL Particle Number	−0.035	0.797	56
LDL-1 Particle Number	−0.012	0.927	56
LDL-2 Particle Number	−0.045	0.740	56
LDL-3 Particle Number	0.005	0.972	56
LDL-4 Particle Number	−0.033	0.810	56
LDL-5 Particle Number	−0.009	0.947	56
LDL-6 Particle Number	0.085	0.533	56
VLDL Triglycerides	−0.063	0.646	56
IDL Triglycerides	−0.092	0.501	56
LDL Triglycerides	0.021	0.880	56
HDL Triglycerides	−0.158	0.245	56
VLDL Cholesterol	−0.126	0.355	56
IDL Cholesterol	−0.043	0.755	56
VLDL Free Cholesterol	−0.134	0.323	56
IDL Free Cholesterol	−0.050	0.716	56
LDL Free Cholesterol	−0.028	0.838	56
HDL Free Cholesterol	0.120	0.377	56
VLDL Phospholipids	−0.089	0.514	56
IDL Phospholipids	−0.036	0.791	56
LDL Phospholipids	−0.051	0.707	56
HDL Phospholipids	0.086	0.527	56
HDL Apo-A1	0.077	0.571	56
HDL Apo-A2	0.173	0.202	56
VLDL Apo-B	−0.115	0.398	56
IDL Apo-B	−0.058	0.671	56
LDL Apo-B	−0.035	0.797	56
VLDL-1 Triglycerides	−0.063	0.646	56
VLDL-2 Triglycerides	−0.032	0.817	56
VLDL-3 Triglycerides	−0.045	0.740	56
VLDL-4 Triglycerides	−0.110	0.418	56
VLDL-5 Triglycerides	−0.190	0.161	56
VLDL-1 Cholesterol	−0.095	0.484	56
VLDL-2 Cholesterol	−0.089	0.517	56
VLDL-3 Cholesterol	−0.051	0.709	56
VLDL-4 Cholesterol	−0.125	0.358	56
VLDL-5 Cholesterol	−.293*	0.028	56
VLDL-1 Free Cholesterol	−0.085	0.535	56
VLDL-2 Free Cholesterol	−0.096	0.480	56
VLDL-3 Free Cholesterol	−0.072	0.598	56
VLDL-4 Free Cholesterol	−0.133	0.329	56
VLDL-5 Free Cholesterol	−0.229	0.090	56
VLDL-1 Phospholipids	−0.066	0.629	56
VLDL-2 Phospholipids	−0.027	0.842	56
VLDL-3 Phospholipids	−0.034	0.804	56
VLDL-4 Phospholipids	−0.152	0.265	56
VLDL-5 Phospholipids	−0.263	0.050	56
LDL-1 Triglycerides	−0.072	0.600	56
LDL-2 Triglycerides	−0.067	0.621	56
LDL-3 Triglycerides	−0.138	0.312	56
LDL-4 Triglycerides	−0.057	0.676	56
LDL-5 Triglycerides	−0.029	0.834	56
LDL-6 Triglycerides	0.156	0.250	56
LDL-1 Cholesterol	−0.053	0.699	56
LDL-2 Cholesterol	−0.034	0.803	56
LDL-3 Cholesterol	0.004	0.976	56
LDL-4 Cholesterol	−0.039	0.773	56
LDL-5 Cholesterol	−0.006	0.964	56
LDL-6 Cholesterol	0.108	0.427	56
LDL-1 Free Cholesterol	−0.035	0.797	56
LDL-2 Free Cholesterol	−0.009	0.948	56
LDL-3 Free Cholesterol	0.039	0.777	56
LDL-4 Free Cholesterol	0.008	0.951	56
LDL-5 Free Cholesterol	0.024	0.863	56
LDL-6 Free Cholesterol	0.147	0.281	56
LDL-1 Phospholipids	−0.064	0.637	56
LDL-2 Phospholipids	−0.029	0.832	56
LDL-3 Phospholipids	0.003	0.980	56
LDL-4 Phospholipids	−0.042	0.761	56
LDL-5 Phospholipids	−0.013	0.927	56
LDL-6 Phospholipids	0.102	0.454	56
LDL-1 Apo-B	−0.015	0.913	56
LDL-2 Apo-B	−0.043	0.751	56
LDL-3 Apo-B	0.005	0.972	56
LDL-4 Apo-B	−0.033	0.809	56
LDL-5 Apo-B	−0.009	0.947	56
LDL-6 Apo-B	0.085	0.533	56
HDL-1 Triglycerides	−0.158	0.245	56
HDL-2 Triglycerides	−0.173	0.202	56
HDL-3 Triglycerides	−0.111	0.417	56
HDL-4 Triglycerides	−0.153	0.261	56
HDL-1 Cholesterol	0.076	0.580	56
HDL-2 Cholesterol	−0.002	0.991	56
HDL-3 Cholesterol	0.160	0.239	56
HDL-4 Cholesterol	0.127	0.351	56
HDL-1 Free Cholesterol	0.100	0.464	56
HDL-2 Free Cholesterol	0.161	0.235	56
HDL-3 Free Cholesterol	0.150	0.271	56
HDL-4 Free Cholesterol	0.196	0.147	56
HDL-1 Phospholipids	0.050	0.712	56
HDL-2 Phospholipids	0.008	0.956	56
HDL-3 Phospholipids	0.159	0.241	56
HDL-4 Phospholipids	0.122	0.372	56
HDL-1 Apo-A1	−0.055	0.688	56
HDL-2 Apo-A1	0.011	0.935	56
HDL-3 Apo-A1	0.099	0.470	56
HDL-4 Apo-A1	0.106	0.436	56
HDL-1 Apo-A2	0.045	0.742	56
HDL-2 Apo-A2	0.095	0.487	56
HDL-3 Apo-A2	0.161	0.235	56
HDL-4 Apo-A2	0.098	0.472	56

**Table:** The estimated regression coefficient from the least absolute shrinkage and selection operator method with percentage of models from the 1000 bootstrap samples that include each of the lipoprotein subfractions.

**Lipoprotein subfractions**	**Estimated regression coefficient**	**Percent included**
Lipoprotein a	56.96	78.1
Total Plasma Apo-A1	0	18.6
Total Plasma Apo-A2	0	5.9
Total Plasma Apo-B	0	0.8
VLDL Particle Number	0	4.5
IDL Particle Number	0	3.7
LDL Particle Number	0	0.2
LDL-1 Particle Number	0	1.2
LDL-2 Particle Number	0	11.7
LDL-3 Particle Number	0	4
LDL-4 Particle Number	0	2.5
LDL-5 Particle Number	0	0.8
LDL-6 Particle Number	0	14.4
VLDL Triglycerides	0	0.3
IDL Triglycerides	0	5.4
LDL Triglycerides	0	12.8
HDL Triglycerides	−3.132	44.3
VLDL Cholesterol	0	0.8
IDL Cholesterol	0	4.7
VLDL Free Cholesterol	0	2.1
IDL Free Cholesterol	0	5.1
LDL Free Cholesterol	0	1.5
HDL Free Cholesterol	0	27.3
VLDL Phospholipids	0	0.2
IDL Phospholipids	0	1.3
LDL Phospholipids	0	0.9
HDL Phospholipids	0	2.5
HDL Apo-A1	0	6.2
HDL Apo-A2	0	6.9
VLDL Apo-B	0	3.4
IDL Apo-B	0	1.7
LDL Apo-B	0	0.1
VLDL-1 Triglycerides	0	7.9
VLDL-2 Triglycerides	0	2.8
VLDL-3 Triglycerides	0	2.3
VLDL-4 Triglycerides	0	5.2
VLDL-5 Triglycerides	0	31.3
VLDL-1 Cholesterol	0	3.5
VLDL-2 Cholesterol	0	4.9
VLDL-3 Cholesterol	0	7
VLDL-4 Cholesterol	0	9.6
VLDL-5 Cholesterol	–32.512	54.3
VLDL-1 Free Cholesterol	0	3.1
VLDL-2 Free Cholesterol	0	5.2
VLDL-3 Free Cholesterol	0	0.4
VLDL-4 Free Cholesterol	0	1.6
VLDL-5 Free Cholesterol	0	23.6
VLDL-1 Phospholipids	0	2.3
VLDL-2 Phospholipids	0	1
VLDL-3 Phospholipids	0	4.6
VLDL-4 Phospholipids	0	4.1
VLDL-5 Phospholipids	−78.469	58.8
LDL-1 Triglycerides	0	21.9
LDL-2 Triglycerides	0	22.4
LDL-3 Triglycerides	0	12.4
LDL-4 Triglycerides	0	18.9
LDL-5 Triglycerides	0	8.4
LDL-6 Triglycerides	0	27.7
LDL-1 Cholesterol	0	24.3
LDL-2 Cholesterol	0	11
LDL-3 Cholesterol	0	15.8
LDL-4 Cholesterol	0	2.9
LDL-5 Cholesterol	0	4.8
LDL-6 Cholesterol	0	3.2
LDL-1 Free Cholesterol	0	7.4
LDL-2 Free Cholesterol	0	15.2
LDL-3 Free Cholesterol	0	4.8
LDL-4 Free Cholesterol	0	4.1
LDL-5 Free Cholesterol	0	3.8
LDL-6 Free Cholesterol	21.949	51.6
LDL-1 Phospholipids	0	3.2
LDL-2 Phospholipids	−3.317	18.8
LDL-3 Phospholipids	−2.553	21.2
LDL-4 Phospholipids	−6.362	26.5
LDL-5 Phospholipids	0	5.2
LDL-6 Phospholipids	0	0.7
LDL-1 Apo-B	0	5
LDL-2 Apo-B	0	9.9
LDL-3 Apo-B	0	4
LDL-4 Apo-B	0	2.4
LDL-5 Apo-B	0	0.7
LDL-6 Apo-B	0	13.2
HDL-1 Triglycerides	−26.616	31.5
HDL-2 Triglycerides	0	11.4
HDL-3 Triglycerides	0	7.5
HDL-4 Triglycerides	0	31.7
HDL-1 Cholesterol	0	15.4
HDL-2 Cholesterol	0	1.4
HDL-3 Cholesterol	18.396	39.5
HDL-4 Cholesterol	0	11.6
HDL-1 Free Cholesterol	0	5.7
HDL-2 Free Cholesterol	15.901	33.8
HDL-3 Free Cholesterol	0	14.9
HDL-4 Free Cholesterol	36.547	74.3
HDL-1 Phospholipids	0	5.9
HDL-2 Phospholipids	0	3.2
HDL-3 Phospholipids	0	13.9
HDL-4 Phospholipids	0	9
HDL-1 Apo-A1	0	13.2
HDL-2 Apo-A1	0	17.4
HDL-3 Apo-A1	0	7.6
HDL-4 Apo-A1	0	4
HDL-1 Apo-A2	0	18.4
HDL-2 Apo-A2	0	6.2
HDL-3 Apo-A2	0	14
HDL-4 Apo-A2	0	2.5

**Table:** The estimated regression coefficient from the least absolute shrinkage and selection operator method with percentage of models from the 1000 bootstrap samples that include each of the lipoprotein subfractions and CVD risk factors.

**Lipid variables and CVD risk factors**	**Estimated regression coefficient**	**Percent included**
Age	0	49.7
Hyperkolesterol	0	53.2
Hypertension, medically treated	−49.497	91.7
Diabetes mellitus	0	43
Previous CVD	0	66
Hereditary CVD	0	61.9
Body mass index	0	39.3
Smoking	0	48.4
Total plasma Triglycerides	0	0
Total plasma Cholesterol	0	2.5
LDL-C	0	1.3
HDL-C	0	3.7
Total Plasma Apo-A1	0	11.6
Total Plasma Apo-A2	0	5.3
Total Plasma Apo-B	0	0.3
Apo-B/Apo-A1	0	2.1
LDL-C/HDL-C	0	1.2
Lipoprotein a	0	67.1
Total Plasma Particle Number	0	0.3
VLDL Particle Number	0	0.4
IDL Particle Number	0	2.8
LDL Particle Number	0	0
LDL-1 Particle Number	0	7
LDL-2 Particle Number	0	5.8
LDL-3 Particle Number	0	1.2
LDL-4 Particle Number	0	1.7
LDL-5 Particle Number	0	0.1
LDL-6 Particle Number	0	7.4
VLDL Triglycerides	0	0
IDL Triglycerides	0	5.1
LDL Triglycerides	0	19.8
HDL Triglycerides	0	25
VLDL Cholesterol	0	0.7
IDL Cholesterol	0	3.7
VLDL Free Cholesterol	0	0
IDL Free Cholesterol	0	2.5
LDL Free Cholesterol	0	0.8
HDL Free Cholesterol	0	14.6
VLDL Phospholipids	0	1.6
IDL Phospholipids	0	1.2
LDL Phospholipids	0	0.1
HDL Phospholipids	0	0.9
HDL Apo-A1	0	5
HDL Apo-A2	0	5.7
VLDL Apo-B	0	0.7
IDL Apo-B	0	1.2
LDL Apo-B	0	0
VLDL-1 Triglycerides	0	6.3
VLDL-2 Triglycerides	0	1.9
VLDL-3 Triglycerides	0	1.6
VLDL-4 Triglycerides	0	7.4
VLDL-5 Triglycerides	0	16.3
VLDL-1 Cholesterol	0	1.5
VLDL-2 Cholesterol	0	4
VLDL-3 Cholesterol	0	5
VLDL-4 Cholesterol	0	6.2
VLDL-5 Cholesterol	0	3.5
VLDL-1 Free Cholesterol	0	1.9
VLDL-2 Free Cholesterol	0	2.5
VLDL-3 Free Cholesterol	0	0.1
VLDL-4 Free Cholesterol	0	4.6
VLDL-5 Free Cholesterol	0	25.9
VLDL-1 Phospholipids	0	7.2
VLDL-2 Phospholipids	0	0.6
VLDL-3 Phospholipids	0	5.9
VLDL-4 Phospholipids	0	4.4
VLDL-5 Phospholipids	0	44.8
LDL-1 Triglycerides	0	2.4
LDL-2 Triglycerides	0	9.4
LDL-3 Triglycerides	0	8.5
LDL-4 Triglycerides	0	18.6
LDL-5 Triglycerides	0	5.7
LDL-6 Triglycerides	0	26.1
LDL-1 Cholesterol	0	23.4
LDL-2 Cholesterol	0	8.5
LDL-3 Cholesterol	0	2.4
LDL-4 Cholesterol	0	1.3
LDL-5 Cholesterol	0	2.3
LDL-6 Cholesterol	0	1.2
LDL-1 Free Cholesterol	0	2.9
LDL-2 Free Cholesterol	0	16.3
LDL-3 Free Cholesterol	0	4
LDL-4 Free Cholesterol	0	1.2
LDL-5 Free Cholesterol	0	5.8
LDL-6 Free Cholesterol	0	55.4
LDL-1 Phospholipids	0	3
LDL-2 Phospholipids	0	9.8
LDL-3 Phospholipids	0	12.7
LDL-4 Phospholipids	0	21.3
LDL-5 Phospholipids	0	2.1
LDL-6 Phospholipids	0	0.2
LDL-1 Apo-B	0	4.4
LDL-2 Apo-B	0	5.7
LDL-3 Apo-B	0	0.9
LDL-4 Apo-B	0	1.6
LDL-5 Apo-B	0	0.1
LDL-6 Apo-B	0	7.2
HDL-1 Triglycerides	0	26.9
HDL-2 Triglycerides	0	6
HDL-3 Triglycerides	0	1.7
HDL-4 Triglycerides	0	28.2
HDL-1 Cholesterol	0	17.8
HDL-2 Cholesterol	0	4.6
HDL-3 Cholesterol	0	32.2
HDL-4 Cholesterol	0	6.7
HDL-1 Free Cholesterol	0	8.9
HDL-2 Free Cholesterol	0	28.6
HDL-3 Free Cholesterol	0	14.7
HDL-4 Free Cholesterol	0	44.6
HDL-1 Phospholipids	0	5.6
HDL-2 Phospholipids	0	2.9
HDL-3 Phospholipids	0	27.7
HDL-4 Phospholipids	0	2.9
HDL-1 Apo-A1	0	1.5
HDL-2 Apo-A1	0	1.2
HDL-3 Apo-A1	0	4.2
HDL-4 Apo-A1	0	3
HDL-1 Apo-A2	0	23.8
HDL-2 Apo-A2	0	4.6
HDL-3 Apo-A2	0	9.7
HDL-4 Apo-A2	0	2.3

## Appendix 1 .

LDL, low-density lipoprotein; HDL, high-density lipoprotein; Apo-A1, apolipoprotein A1; Apo-A2, apolipoprotein A2; Apo-B, apolipoprotein B; VLDL, very-low-density lipoprotein; IDL, intermediate-density lipoprotein. *Included as a CVD risk factor in the statistical analyses, †Duplicate, removed from all statistical analyses.

## Appendix 2 .

LDL, low-density lipoprotein; HDL, high-density lipoprotein; VLDL, very-low-density lipoprotein; IDL, intermediate-density lipoprotein. *The density ranges for VLDL subfractions 1–5 are specified in *Lindgren FT, Jensen LL, Hatch FT (1972). The isolation and quantitative analysis of serum lipoproteins. In Nelson GJ (ed.) Blood lipids and lipoproteins: Quantitation, composition and metabolism. Wiley-Interscience, New York, p 181*–*274.*

## Appendix 3 . Flowchart of the enrollment.

* Vesterbekkmo, E. K., Madssen, E., Aamot Aksetøy, I. L., Follestad, T., Nilsen, H. O., Hegbom, K.,Wisløff, U., & Wiseth, R. (2022). CENIT (Impact of Cardiac Exercise Training on Lipid Content in Coronary Atheromatous Plaques Evaluated by Near-Infrared Spectroscopy): A Randomized Trial. *J Am Heart Assoc*, *11*(10), e024705. https://doi.org/10.1161/jaha.121.024705.

## Appendix 4 .

NMR, nuclear magnetic resonance; HDL-C, high-density lipoprotein cholesterol; LDL-C, low-density lipoprotein cholesterol.

## Appendix 5 .

LDL, low-density lipoprotein; HDL, high-density lipoprotein; Apo-A1, apolipoprotein A1; Apo-A2, apolipoprotein A2; Apo-B, apolipoprotein B; VLDL, very-low-density lipoprotein; IDL, intermediate-density lipoprotein.

## Appendix 6 .

LDL, low-density lipoprotein; HDL, high-density lipoprotein; Apo-A1, apolipoprotein A1; Apo-A2, apolipoprotein A2; Apo-B, apolipoprotein B; VLDL, very-low-density lipoprotein; IDL, intermediate-density lipoprotein.

## Appendix 7 .

CVD, cardiovascular disease; LDL, low-density lipoprotein; LDL-C, low-density lipoprotein cholesterol; HDL, high-density lipoprotein; HDL-C, high-density lipoprotein cholesterol; Apo-A1, apolipoprotein A1; Apo-A2, apolipoprotein A2; Apo-B, apolipoprotein B; VLDL, very-low-density lipoprotein; IDL, intermediate-density lipoprotein.

## References

[1] Björkegren J.L.M., Lusis A.J. (2022). Atherosclerosis: Recent developments. Cell.

[2] Virmani R., Burke A.P., Kolodgie F.D., Farb A. (2003). Pathology of the thin-cap fibroatheroma: a type of vulnerable plaque. J. Interv. Cardiol..

[3] Gardner C.M., Tan H., Hull E.L., Lisauskas J.B., Sum S.T., Meese T.M., Jiang C., Madden S.P., Caplan J.D., Burke A.P., Virmani R., Goldstein J., Muller J.E. (2008). Detection of lipid core coronary plaques in autopsy specimens with a novel catheter-based near-infrared spectroscopy system. J. Am. Coll. Cardiol. Img..

[4] Waxman S., Dixon S.R., L'Allier P., Moses J.W., Petersen J.L., Cutlip D., Tardif J.C., Nesto R.W., Muller J.E., Hendricks M.J., Sum S.T., Gardner C.M., Goldstein J.A., Stone G.W., Krucoff M.W. (2009). In vivo validation of a catheter-based near-infrared spectroscopy system for detection of lipid core coronary plaques: initial results of the SPECTACL study. J. Am. Coll. Cardiol. Img..

[5] Erlinge D., Maehara A., Ben-Yehuda O., Bøtker H.E., Maeng M., Kjøller-Hansen L., Engstrøm T., Matsumura M., Crowley A., Dressler O., Mintz G.S., Fröbert O., Persson J., Wiseth R., Larsen A.I., Okkels Jensen L., Nordrehaug J.E., Bleie Ø., Omerovic E., Stone G.W. (2021). Identification of vulnerable plaques and patients by intracoronary near-infrared spectroscopy and ultrasound (PROSPECT II): a prospective natural history study. Lancet.

[6] Madder R.D., Husaini M., Davis A.T., VanOosterhout S., Khan M., Wohns D., McNamara R.F., Wolschleger K., Gribar J., Collins J.S., Jacoby M., Decker J.M., Hendricks M., Sum S.T., Madden S., Ware J.H., Muller J.E. (2016). Large lipid-rich coronary plaques detected by near-infrared spectroscopy at non-stented sites in the target artery identify patients likely to experience future major adverse cardiovascular events. Eur. Heart J. Cardiovasc. Imaging.

[7] Schuurman A.S., Vroegindewey M., Kardys I., Oemrawsingh R.M., Cheng J.M., de Boer S., Garcia-Garcia H.M., van Geuns R.J., Regar E.S., Daemen J., van Mieghem N.M., Serruys P.W., Boersma E., Akkerhuis K.M. (2018). Near-infrared spectroscopy-derived lipid core burden index predicts adverse cardiovascular outcome in patients with coronary artery disease during long-term follow-up. Eur. Heart J..

[8] Waksman R., Di Mario C., Torguson R., Ali Z.A., Singh V., Skinner W.H., Artis A.K., Cate T.T., Powers E., Kim C., Regar E., Wong S.C., Lewis S., Wykrzykowska J., Dube S., Kazziha S., van der Ent M., Shah P., Craig P.E., Newby D. (2019). Identification of patients and plaques vulnerable to future coronary events with near-infrared spectroscopy intravascular ultrasound imaging: a prospective, cohort study. Lancet.

[9] Feingold K.R., Feingold K.R., Anawalt B., Boyce A., Chrousos G., de Herder W.W., Dhatariya K., Dungan K., Hershman J.M., Hofland J., Kalra S., Kaltsas G., Koch C., Kopp P., Korbonits M., Kovacs C.S., Kuohung W., Laferrère B., Levy M., McGee E.A., McLachlan R., Morley J.E., New M., Purnell J., Sahay R., Singer F., Sperling M.A., Stratakis C.A., Trence D.L., Wilson D.P. (2000). Endotext.

[10] Soppert J., Lehrke M., Marx N., Jankowski J., Noels H. (2020). Lipoproteins and lipids in cardiovascular disease: from mechanistic insights to therapeutic targeting. Adv. Drug Deliv. Rev..

[11] Domanski M.J., Tian X., Wu C.O., Reis J.P., Dey A.K., Gu Y., Zhao L., Bae S., Liu K., Hasan A.A., Zimrin D., Farkouh M.E., Hong C.C., Lloyd-Jones D.M., Fuster V. (2020). Time course of LDL cholesterol exposure and cardiovascular disease event risk. J. Am. Coll. Cardiol..

[12] Renkens M.P.L., Mintz G.S., Torguson R., Di Mario C., Ten Cate T., Ali Z.A., Singh V., Skinner W., Artis A., Garcia-Garcia H.M., de Winter R.J., Wykrzykowska J.J., Waksman R. (2021). Non-culprit MACE-rate in LRP: The influence of optimal medical therapy using DAPT and statins. Cardiovasc. Revasc. Med..

[13] Sachdeva A., Cannon C.P., Deedwania P.C., Labresh K.A., Smith S.C., Dai D., Hernandez A., Fonarow G.C. (2009). Lipid levels in patients hospitalized with coronary artery disease: an analysis of 136,905 hospitalizations in get with the guidelines. Am. Heart J..

[14] Wong N.D., Zhao Y., Quek R.G.W., Blumenthal R.S., Budoff M.J., Cushman M., Garg P., Sandfort V., Tsai M., Lopez J.A.G. (2017). Residual atherosclerotic cardiovascular disease risk in statin-treated adults: The multi-ethnic study of atherosclerosis. J. Clin. Lipidol..

[15] Hoogeveen R.C., Gaubatz J.W., Sun W., Dodge R.C., Crosby J.R., Jiang J., Couper D., Virani S.S., Kathiresan S., Boerwinkle E., Ballantyne C.M. (2014). Small dense low-density lipoprotein-cholesterol concentrations predict risk for coronary heart disease: the atherosclerosis risk in communities (ARIC) study. Arterioscler. Thromb. Vasc. Biol..

[16] Li J.J., Zhang Y., Li S., Cui C.J., Zhu C.G., Guo Y.L., Wu N.Q., Xu R.X., Liu G., Dong Q., Sun J. (2016). Large HDL subfraction but not HDL-C is closely linked with risk factors, coronary severity and outcomes in a cohort of nontreated patients with stable coronary artery disease: A prospective observational study. Medicine (Baltimore).

[17] Vekic J., Zeljkovic A., Cicero A.F.G., Janez A., Stoian A.P., Sonmez A., Rizzo M. (2022). Atherosclerosis development and progression: the role of atherogenic small, dense LDL. Medicina (Kaunas).

[18] Kronenberg F., Mora S., Stroes E.S.G., Ference B.A., Arsenault B.J., Berglund L., Dweck M.R., Koschinsky M., Lambert G., Mach F., McNeal C.J., Moriarty P.M., Natarajan P., Nordestgaard B.G., Parhofer K.G., Virani S.S., von Eckardstein A., Watts G.F., Stock J.K., Catapano A.L. (2022). Lipoprotein(a) in atherosclerotic cardiovascular disease and aortic stenosis: a European Atherosclerosis Society consensus statement. Eur. Heart J..

[19] Rikhi R., Hammoud A., Ashburn N., Snavely A.C., Michos E.D., Chevli P., Tsai M.Y., Herrington D., Shapiro M.D. (2022). Relationship of low-density lipoprotein-cholesterol and lipoprotein(a) to cardiovascular risk: The multi-ethnic study of atherosclerosis (MESA). Atherosclerosis.

[20] Saleheen D., Haycock P.C., Zhao W., Rasheed A., Taleb A., Imran A., Abbas S., Majeed F., Akhtar S., Qamar N., Zaman K.S., Yaqoob Z., Saghir T., Rizvi S.N.H., Memon A., Mallick N.H., Ishaq M., Rasheed S.Z., Memon F.U., Danesh J. (2017). Apolipoprotein(a) isoform size, lipoprotein(a) concentration, and coronary artery disease: a mendelian randomisation analysis. Lancet Diab. Endocrinol..

[21] Dobrolińska M.M., Gąsior P., Wańha W., Pietraszewski P., Pociask E., Smolka G., Wojakowski W., Roleder T. (2021). The influence of high-density lipoprotein cholesterol on maximal lipid core burden indexing thin cap fibrous atheroma lesions as assessed by near infrared spectroscopy. Cardiol. J..

[22] Honda S., Sidharta S.L., Shishikura D., Takata K., Di Giovanni G.A., Nguyen T., Janssan A., Kim S.W., Andrews J., Psaltis P.J., Worthley M.I., Nicholls S.J. (2017). High-density lipoprotein cholesterol associated with change in coronary plaque lipid burden assessed by near infrared spectroscopy. Atherosclerosis.

[23] Nakamura H., Kataoka Y., Nicholls S.J., Puri R., Kitahara S., Murai K., Sawada K., Matama H., Iwai T., Honda S., Fujino M., Takagi K., Yoneda S., Otsuka F., Nishihira K., Asaumi Y., Tsujita K., Noguchi T. (2022). Elevated Lipoprotein(a) as a potential residual risk factor associated with lipid-rich coronary atheroma in patients with type 2 diabetes and coronary artery disease on statin treatment: Insights from the REASSURE-NIRS registry. Atherosclerosis.

[24] Torguson R., Mintz G.S., Zhang C., Case B.C., Di Mario C., Garcia-Garcia H.M., Waksman R. (2022). Lipid-rich plaque density and low-density lipoprotein cholesterol in statin-treated versus statin-na&iuml;ve patients: a post hoc analysis of the LRP study. EuroIntervention.

[25] Vesterbekkmo E.K., Madssen E., Aamot Aksetøy I.L., Follestad T., Nilsen H.O., Hegbom K., Wisløff U., Wiseth R. (2022). CENIT (Impact of cardiac exercise training on lipid content in coronary atheromatous plaques evaluated by near-infrared spectroscopy): a randomized trial. J. Am. Heart Assoc..

[26] Kini A.S., Baber U., Kovacic J.C., Limaye A., Ali Z.A., Sweeny J., Maehara A., Mehran R., Dangas G., Mintz G.S., Fuster V., Narula J., Sharma S.K., Moreno P.R. (2013). Changes in plaque lipid content after short-term intensive versus standard statin therapy: the YELLOW trial (reduction in yellow plaque by aggressive lipid-lowering therapy). J. Am. Coll. Cardiol..

[27] Nawrocki J.W., Weiss S.R., Davidson M.H., Sprecher D.L., Schwartz S.L., Lupien P.J., Jones P.H., Haber H.E., Black D.M. (1995). Reduction of LDL cholesterol by 25% to 60% in patients with primary hypercholesterolemia by atorvastatin, a new HMG-CoA reductase inhibitor. Arterioscler. Thromb. Vasc. Biol..

[28] Jiménez B., Holmes E., Heude C., Tolson R.F., Harvey N., Lodge S.L., Chetwynd A.J., Cannet C., Fang F., Pearce J.T.M., Lewis M.R., Viant M.R., Lindon J.C., Spraul M., Schäfer H., Nicholson J.K. (2018). Quantitative lipoprotein subclass and low molecular weight metabolite analysis in human serum and plasma by (1)H NMR spectroscopy in a multilaboratory trial. Anal. Chem..

[29] Gao X., Starmer J., Martin E.R. (2008). A multiple testing correction method for genetic association studies using correlated single nucleotide polymorphisms. Genet. Epidemiol..

[30] Santos Ferreira D.L., Williams D.M., Kangas A.J., Soininen P., Ala-Korpela M., Smith G.D., Jarvelin M.R., Lawlor D.A. (2017). Association of pre-pregnancy body mass index with offspring metabolic profile: Analyses of 3 European prospective birth cohorts. PLoS Med..

[31] Friedman J., Hastie T., Tibshirani R. (2010). Regularization paths for generalized linear models via coordinate descent. J. Stat. Softw..

[32] Enas E.A., Varkey B., Dharmarajan T.S., Pare G., Bahl V.K. (2019). Lipoprotein(a): An independent, genetic, and causal factor for cardiovascular disease and acute myocardial infarction. Indian Heart J..

[33] Tsimikas S. (2017). A test in context: lipoprotein(a): Diagnosis, prognosis, controversies, and emerging therapies. J. Am. Coll. Cardiol..

[34] Tsimikas S., Fazio S., Ferdinand K.C., Ginsberg H.N., Koschinsky M.L., Marcovina S.M., Moriarty P.M., Rader D.J., Remaley A.T., Reyes-Soffer G., Santos R.D., Thanassoulis G., Witztum J.L., Danthi S., Olive M., Liu L. (2018). NHLBI working group recommendations to reduce lipoprotein(a)-mediated risk of cardiovascular disease and aortic stenosis. J. Am. Coll. Cardiol..

[35] de Boer L.M., Oorthuys A.O.J., Wiegman A., Langendam M.W., Kroon J., Spijker R., Zwinderman A.H., Hutten B.A. (2022). Statin therapy and lipoprotein(a) levels: a systematic review and meta-analysis. Eur. J. Prev. Cardiol..

[36] Tsimikas S., Gordts P., Nora C., Yeang C., Witztum J.L. (2020). Statin therapy increases lipoprotein(a) levels. Eur. Heart J..

[37] Willeit P., Ridker P.M., Nestel P.J., Simes J., Tonkin A.M., Pedersen T.R., Schwartz G.G., Olsson A.G., Colhoun H.M., Kronenberg F., Drechsler C., Wanner C., Mora S., Lesogor A., Tsimikas S. (2018). Baseline and on-statin treatment lipoprotein(a) levels for prediction of cardiovascular events: individual patient-data meta-analysis of statin outcome trials. Lancet.

[38] Handhle A., Viljoen A., Wierzbicki A.S. (2021). Elevated lipoprotein(a): background, current insights and future potential therapies. Vasc. Health Risk Manag..

[39] Niccoli G., Cin D., Scalone G., Panebianco M., Abbolito S., Cosentino N., Jacoangeli F., Refaat H., Gallo G., Salerno G., Volpe M., Crea F., De Biase L. (2016). Lipoprotein (a) is related to coronary atherosclerotic burden and a vulnerable plaque phenotype in angiographically obstructive coronary artery disease. Atherosclerosis.

[40] Landray M.J., Haynes R., Hopewell J.C., Parish S., Aung T., Tomson J., Wallendszus K., Craig M., Jiang L., Collins R., Armitage J. (2014). Effects of extended-release niacin with laropiprant in high-risk patients. N. Engl. J. Med..

[41] Voight B.F., Peloso G.M., Orho-Melander M., Frikke-Schmidt R., Barbalic M., Jensen M.K., Hindy G., Hólm H., Ding E.L., Johnson T., Schunkert H., Samani N.J., Clarke R., Hopewell J.C., Thompson J.F., Li M., Thorleifsson G., Newton-Cheh C., Musunuru K., Kathiresan S. (2012). Plasma HDL cholesterol and risk of myocardial infarction: a mendelian randomisation study. Lancet.

[42] Cupido A.J., Asselbergs F.W., Schmidt A.F., Hovingh G.K. (2022). Low-density lipoprotein cholesterol attributable cardiovascular disease risk is sex specific. J. Am. Heart Assoc..

[43] Robinson G.A., Peng J., Peckham H., Radziszewska A., Butler G., Pineda-Torra I., Jury E.C., Ciurtin C. (2021). Sex hormones drive changes in lipoprotein metabolism. iScience.

